# Acceptable objectives of empirical research in bioethics: a qualitative exploration of researchers’ views

**DOI:** 10.1186/s12910-022-00845-1

**Published:** 2022-12-28

**Authors:** Emilian Mihailov, Veerle Provoost, Tenzin Wangmo

**Affiliations:** 1 Faculty of Philosophy, University of Bucharest, Bucharest, Romania; 2 Bioethics Institute Ghent, University of Ghent, Ghent, Belgium; 3 Institute for Biomedical Ethics, University of Basel, Basel, Switzerland

**Keywords:** Empirical bioethics, Empirical research in bioethics, Moral attitudes, Moral reasoning, Normative recommendations, Source of morality

## Abstract

**Background:**

This is the first qualitative study to investigate how researchers, who do empirical work in bioethics, relate to objectives of empirical research in bioethics (ERiB). We explore reasons that make some objectives more acceptable, while others are deemed less acceptable.

**Methods:**

Using qualitative exploratory study design, we interviewed bioethics researchers, who were selected to represent different types of scholars working in the field. The interview data of 25 participants were analyzed in this paper using thematic analysis.

**Results:**

From the eight objectives presented to the study participants, understanding the context of a bioethical issue and identifying ethical issues in practice received unanimous agreement. Participants also supported other objectives of ERiB but with varying degrees of agreement. The most contested objectives were striving to draw normative recommendations and developing and justifying moral principles. The is-ought gap was not considered an obstacle to ERiB, but rather a warning sign to critically reflect on the normative implications of empirical results.

**Conclusions:**

Our results show that the most contested objectives are also the more ambitious ones, whereas the least contested ones focus on producing empirical results. The potential of empirical research to be useful for bioethics was mostly based on the reasoning pattern that empirical data can provide a testing ground for elements of normative theory. Even though empirical research can inform many parts of bioethical inquiry, normative expertise is recommended to guide ERiB. The acceptability of ambitious objectives for ERiB boils down to finding firm ground for the integration of empirical facts in normative inquiry.

**Supplementary Information:**

The online version contains supplementary material available at 10.1186/s12910-022-00845-1.

## Introduction

Bioethics has transitioned to a field where many disciplines and many methods contribute to solving practical issues [1–11]. However, in light of the is-ought gap, one can question the extent to which empirical research contributes to bioethics [12]. If we should not *draw* ethical prescriptions from facts, then how can empirical research be useful?

Because ethical arguments are entangled with empirical assumptions about stakeholders and conditions of reasoning, bioethics welcomes many potential objectives of empirical research that are relevant for moral questions. For instance, descriptive ethics studies explore stakeholders’ responses to bioethical questions and try explain how people arrive at certain moral opinions and reasoning patterns [13–22]. Empirical research can investigate whether people and healthcare professionals comply with ethical guidelines and how ethical solutions are translated into practice [13–15, 18, 23–29]. Furthermore, empirical research reveals the lived experience of stakeholders [14, 30–34]. The engagement with empirical research may help understand how moral questions are relevant and experienced in practice [7, 18, 35, 36]. Researchers who seek to issue recommendations can draw on empirical findings and utilize methodologies that help them integrate those in normative recommendations [15, 32, 37, 38].

However, some authors claim that social sciences are not just a ‘handmaiden’ that simply documents ‘facts’ that ethicists use in their normative arguments. Empirical work, they say, can be used to develop a critique of ethical concepts and principles [39–41], to contribute to the process of justifying moral principles, and to help determine which moral principles and subsequent policies are more appropriate in given contexts [42–44]. Additionally, Sugarman et al. [45] found an increase in empirical studies which address the topic of ethical theory (broadly construed) such as moral development, moral obligation, ethical analysis, philosophy, feminism and humanism.

Opening bioethics to empirical studies resulted in a debate about what are the appropriate conditions to do empirical research in bioethics (ERiB). The reality of empirical research as it is practiced can inform the debate about what objectives are more palatable and legitimate. In this qualitative study, we investigate how researchers, who do empirical work in bioethics, relate to these proposed objectives of ERiB. Given their experience, we explore which reasons are used to argue for the acceptability of these objectives or their lack of acceptability. Our qualitative exploration, thus, illuminates to what extent theoretical proposals for using empirical research in bioethics match with the views of the scholars who carry out this type of work in practice. These views are instrumental to improve interdisciplinary dialogue between empirically and normatively oriented researchers and facilitate reflection on the value and challenges of ERiB.

## Methods

### Interview guide

We developed an interview guide for the overall project, within which we operationalized proposals for using ERiB into eight statements. We asked participants what they thought about the possible objectives and how they viewed their work in relation to the eight objectives that we developed (see interview guide in supplementary file).

In finalizing the 8 objectives, we were guided by the idea of a continuum that starts with focusing on more empirical objectives (with the exploration of the context as the first goal) and builds towards objectives with a direct impact on the normative. Our list of objectives starts with modest expectations towards the contribution of empirical research in the overall bioethical endeavor and moving towards a high ambition. We arrived at a different list from other classifications of ERiB because we tried to strike a balance between feasibility and covering diversity (see [14, 15, 18, 42]). While some objectives of empirical research were treated distinctively in the literature, we decided to put them in the same category. For example, Sulmasy and Sugarman [14] conceptualized the goal of testing norms and assessing likely consequences as distinct. Indeed, respecting norms and calculating consequences map different evaluative mindsets. From the perspective of using ERiB, it seems to us that a more general category can include both, namely evaluating how an ethical recommendation plays out in practice. By contrast with Kon [15], who placed the analysis of empirical findings to recommend changes only in specific ethical norms, we introduced levels of generality. Thus, we asked participants about recommending changes in ethical norms (e) and recommending changes in general principles (f). The rationale for introducing levels of generality is to test researchers’ reactions to how greatly can the empirical data change existing moral principles. With the last objective (h), we invited the participants to reflect upon the goal of using empirical research as a source of morality. This goal was not explicitly present in the literature, but it builds on ambitious use of empirical research to help look for inspiring new sources of morality [17, 18, 42, 43, 46]. We thus wanted to examine how the participants would react to one of the most ambitious objectives of ERiB. We are not claiming that this is an exhaustive list, nor do we claim that any or all these objectives are legitimate. No doubt, some are controversial, but this is what we wanted. By presenting these options, we gave participants the open question to consider how controversial and how legitimate proposed objectives of ERiB are.

### Participant sampling

In order to obtain an unbiased heterogeneous sample of researchers doing ERiB, we used the following search strategy. First, we performed a systematic search to look for article publication in these two sub-fields using two databases – PubMed and SCOPUS. This process allowed us to populate our sample pool. These two databases were selected because they give access to many bioethics publications. For the two databases, we used the following search terms (see Table 1). We placed a time limit of 5 years (01.01.2015–27.02.2020), to increase the likelihood of the researchers still being reachable via the corresponding e-mail address available in the published work.

**Table 1:** Search terms and search outputs

Search terms used	(((((((("Empirical Bioethics") OR "Empirical Ethics") OR "Interdisciplinary Ethics") OR "Interdisciplinary Bioethics") OR "Interdisciplinary Empirical Ethics") OR "empirical-normative") OR "normative-empirical") OR “Empirical research in Bioethics”)
Results PubMed	N = 159
Results Scopus	N = 175

### Search outcome and participant selection

The search resulted in a total of 334 titles, which decreased to 243 after removing duplicates. EM and TW studied the titles and abstracts of all the 243 results, and decided to disregard 52 titles (reasons: 3 articles were from the authors of this paper and 49 were book chapters, which we excluded as we wanted to concentrate our efforts on recruiting authors of peer-reviewed publications). This meant that our sample pool from the two databases was now 191 published peer-reviewed articles. To this, we added 13 papers from other searches that we carried out in Google Scholar for publications in ERiB, resulting in 204 total included results. We made this additional search because we wanted to see if the initial search missed relevant important work. EM classified the 204 into three categories based on the reading of the title and abstract: (a) Empirical: 94; (b) Methodological: 74; and (c) Empirical-argumentative: 36. These three categories were ordered alphabetically to allow simple random selection. We agreed to this selection method to avoid biases associated with interviewing experts in the field. Doing so would have been straightforward in some sense since who is an expert is easy enough to identify as this topic in bioethics is populated by countable number of experts. We decided against expert interviews as it would exclude legitimate voices of “non-experts” but enthusiasts who are working in the field or those who wish to work in this field.

We performed two rounds of selection within each of these three categories. The first selection was carried out in March 2020, where we randomly (simple random) selected 18 participants each from (a) and (b), and 12 from (c). The first authors of these 48 selected titles received emails to participate in our study, and all non-responders received one reminder. During the second round of selection in June 2020, we sought to balance out the proportion of selected participants to capture more female participants. A total of 37 titles were randomly selected representing 19 from group (a), 12 from group (b) and 6 from group (c). The first author of these 37 manuscripts received our request to participate in the study and one reminder was sent to non-responders.

In sum, a total of 85 first authors randomly selected from the extracted titles received our request to participate in the study, of which 24 agreed to participate. This represented a response rate of 28%. To obtain a more diverse sample, upon suggestion of a participant, we recruited two participants that our participant recommended. Of the total 26 participants, 14 were female whereas 12 were male. By experience, 17 were senior researchers and 9 were junior researchers. We further categorized them based on their description of their work and experience as follows: empirical ethicists (8), social scientists working on ethical topics (6), empirical researchers in ethics (11) and theoretical ethicist (1). By geographical location, participants were from the following regions: North America (7), South America (1), Western Europe (14), Asia (2), and Australia (2).

### Data collection

The first author sent each prospective participant an email informing them about the study, its purpose, the researchers, and the voluntary nature of the study. No incentives were offered. Upon receiving an affirmative response, an interview date was scheduled. The interviews were carried using Zoom in light of the Covid-19 pandemic and of the geographical dispersion of the researchers. All interviews were carried out between April 2020 and January 2021. The interviews were recorded upon consent of the participants. They were between 45 and 90 min long, with an average of 60 min. The first author, who was the interviewer, met most of the participants for the first time and there was no prior relationship with them. Only in a few cases, it turned out *per accidens* that he knew the participant in light of prior collaborations or having met them at conferences.

### Data analysis

Audio recordings of each interview were transcribed verbatim by research assistants. During the transcription process, identifying information of the participants were removed. Data analysis proceeded in several steps. To familiarize ourselves with the data, EM and TW read all transcripts and shared their notes on each of them. Thereafter, the entire dataset was coded inductively guided by thematic analysis [47]. Based on the richness of the data, for this paper, we focused on presenting the findings related to the data belonging to eight statements concerning the objectives of ERiB. Using coded materials already available, we further analyzed each statement of the study participant and first grouped these statements into agreement, disagreement and unsure. Based on the quality of responses, the authors decided to classify the unsure responses into either disagreement or agreement based on the nature of the rationale provided. However, we also wanted to keep the nuanced level of such agreement or disagreement, and specific those as such in the findings (see results). Thereafter, we delved into the reasons that participants’ provided for their positions, which were grouped together into different meaning sets. During this process, we decided to exclude one participant from further analysis as the participant did not respond to these statements. Hence, for this paper, data from 25 participants was used. The analysis was checked and validated by all authors to ensure that we could reach a shared interpretation.

## Results

All participants accepted the objectives of (A) understanding the context and (B) identifying ethical issues in practice. The highest number of participants who disagreed was found for two statements concerning drawing normative recommendations (E), and developing and justifying moral principles (F). We present a count of agreements versus disagreements in Table 2 to provide an overview of their attitudes towards the eight statements.

**Table 2:** Participants’ (N = 25) acceptability of the eight objectives of ERiB

Statements	Agree	Disagree
A. Understand the context of the phenomenon under study	25	0
B. Identify ethical issues in practice	25	0
C. Find actual moral attitudes and reasoning patterns relevant to a practice	18	7
D. Evaluate how an ethical recommendation has been implemented	21	4
E. Draw normative recommendations	16	9
F. Develop and justify moral principles	15	10
G. Identify theoretical ethical issues	16*	8
H. Source of morality to build new normative principles, rules or regulations	18	7

*Statement skipped by error

### Understanding the context of the phenomenon under study

All participants agreed that “to understand the context of the phenomenon under study” is an acceptable objective of ERiB. Understanding the context was considered necessary for producing robust practical knowledge about stakeholders’ perspectives and factors that influence ethical decision making: “you need a descriptive account of what is going on and what are the reasons behind the preferences of the patient. Ideally you have 360 degree”. (P20, empirical researcher in ethics) Participants highlighted that the application of ethical principles may backfire if you disregard the context: “You have to contextualize the normative work. Otherwise it's so general and irrelevant, that no one is ever going to take it seriously in the policy world.” (P7, empirical researcher in ethics) Further, several participants underlined that it is an essential starting point towards the overall goal of the research, “I think when you LOOK at lots of empirical bioethics methodologies that have been published in some way or form, that is the FIRST step for all of them.” (P22, empirical ethicist).

### Identifying ethical issues in practice

All participants accepted this objective of ERiB. Some participants considered it to be contingent. They thought that first you have to decide on what ethical issue to focus before starting any empirical research. So, identifying new ethical issues was not the core of empirical research, but a spin off from the primary research question. In this sense, instead of being considered a main objective, this type of output was seen as secondary.

“I think it'd be strange to have a bioethics project, an empirical bioethics project without an ethical issue in mind and you will just go and identify issues. I think that'd be quite strange. Ehm...I think, you'd have an issue in mind. But what DOES happen is you might identify NEW ethical issues, that you never conceived of before, as the project progresses.” (P7, empirical researcher in ethics)

To other participants, this purpose illustrated a bottom-up approach to bioethics in which you are open to what constitutes an ethical issue. One may discover that some issues are more important than others: “And when we GO to the field, we see that these ethical issues are NOT relevant…or there are more ethical issues…or there are ethical issues of MORE IMPORTANCE…in the field.” (P17, social scientist working on ethical topic) The bottom-up approach of identifying new ethical issues was faced with the methodological challenge of who defines what an ethical issue is: "Who identifies ethical issues as 'ethical issues'?. The researcher? The participant? I mean, who gets to identify those? And I think that's a bit of research that needs to be done or discussed.” (P18, social scientist working on ethical topic).

### Finding actual moral attitudes and reasoning patterns

The majority of participants agreed that empirical research could allow researchers “to find actual moral attitudes and reasoning patterns relevant to a practice”. They underlined that many studies in bioethics include such a purpose, “… there are many studies which go along these lines, quantitative surveys on moral attitudes regarding many ethical issues. So, I would guess that the majority of studies in empirical bioethics could be subsumed under this heading.” (P11, empirical ethicist) Some participants felt that this aim also favored a bottom-up approach that could challenge ethical theory: “we should be very interested in what their [people studied] moral attitudes are and how they reason about these cases, not just the intuitions of people who are far removed from the circumstances and thinking only in abstract terms.” (P9, empirical ethicist).

Several participants disagreed with the statement claiming that this objective is merely descriptive. In fact, they laid the boundary between empirical bioethics and sociology:

“If you just say: No, no, I'm just interested in finding out what moral attitudes and reasoning there are, but I will not go further into discussion this ethically. … Then I would say, this goes more into the direction of sociological research and I would say, ok, I wouldn't directly see what the bioethics part of that is.” (P2, empirical ethicist)

Critical voices admitted that such studies could become an important part of empirical bioethics if the researcher engages normatively with the results (at a later point) or uses them for an ethical purpose: “So I don't think this in and of itself is empirical ethics, I think that would just be a descriptive endeavor. But I think that is an important data point within an empirical normative effort.” (P8, social scientist working on ethical topic) One participant suggested that a focus on reasoning patterns—rather than attitudes—was a potential objective of an empirical project because they are more normatively salient than moral attitudes: “to me that's [moral attitudes] more descriptive and contributes less. Reasoning patterns is more, valuable, in my opinion. To me, these are two different things.” (P16, social scientist working on ethical topic).

### Evaluating the implementation of ethical recommendation using empirical research

Twenty one participants considered the evaluation of the implementation of an ethical recommendation to be an acceptable objective of ERiB. They noted that most often researchers could not anticipate the complexities of a clinical context, and hence they needed to see how recommendations play out in the real world: “from the perspective of applied ethics this is even the most important part to make sure that the recommendations become powerful in practice and are accepted and known and learned by those for whom it is important.” (P11, empirical ethicist) One participant considered it similar to other interdisciplinary fields like sociology of law: “I think this is a very important research. … I think sociologists of law do something very similar so they examine whether people adopt to new regulations or not and under which conditions.” (P14, empirical ethicist).

The few participants, who were uncertain about this objective, said that it is not clear whether such studies belong to bioethics. One participant was in doubt about whether this is an actual objective of empirical bioethics, assuming that empirical bioethics is a distinctive project compared to empirical research carried out on bioethics topics: “I do think this is an important role of empirical research in bioethics. Again, I want to say, maybe not, we don’t necessarily want to call it empirical bioethics.” (P6, empirical ethicist) Others considered that it belongs to quality assessment and program evaluation. One rationale for skeptical views was that such kind of research does not depend on ethical expertise:

“If you say, it's really just looking at ‘is this working or is this not working’, then I would again say, what's the bioethics part exactly? Of course you are looking at ethical recommendation, but to be honest … you don't need a bioethicist for doing this. You don't even need ethics expertise to do this kind of research. You could just look at, ok, there is recommendation A, written in the codes or something like that. You can look, is this followed by in the practice or not or something like that.” (P2, empirical ethicist)

### Drawing normative recommendations from empirical research

16 participants thought that a possible objective of ERiB was to make normative recommendations. This group of participants felt that this was the *raison d'être* of empirical research in bioethics: “all empirical work tends to do that, that piece of drawing normative recommendations … that's really why I am in this area.” (P8, social scientist working on ethical topic) One participant was attracted to this objective because it allowed making recommendations that work for practice:

“… in my research of - you know - we went in expecting to find ways to support staff and really, what we found because we realized the moral attitudes and reasoning patterns in that CONTEXT was the normative recommendations were very DIFFERENT. (…) So I think normative recommendations look very different when they're influenced by empirical ethics [than] that they would if they're influenced from more the philosophical top-down theorizing that we typically see, in bioethics.” (P21, empirical researcher in ethics)

Another participant accepted that empirical research could help in drawing normative recommendations by invoking a process of theory testing:

“you can have a normative idea about surrogate decision making and then you could study that question and find that … what we have accepted is not actually true and then you might need to draw a new normative recommendation based on your quantitative evidence ... And that is whole point of science, isn’t it? That we think things are true and then we actually test the hypothesis and we realize oh no. So, to me normative bioethics is like a theory.” (P10, empirical researcher in ethics)

There were participants who conditionally agreed to the statement. They underscored that it is not possible to go directly from empirical results to normative recommendations, even though they thought (sometimes wholeheartedly) that this objective was important. Participant (P22, empirical ethicist) put this thought succinctly as “I’d say yes. But only with a bridging theory or some kind of account of how you use the data to draw normative conclusions”.

A few participants disagreeing to this objective were uncertain that empirical work could help to (e.g. by adding information) draw normative recommendations and it requires an ethicist to do that work. One such participant reported:

“I think it is difficult just based on the data to draw normative recommendations, and actually I think that’s one important thing where the professional, the ethicist comes into play. This is the ethicist’s duty, their job, it’s the ethicist’s job to draw those normative recommendations and not to have that done by the data or by the people who have been interviewed or observed or whatever.” (P5, empirical researcher in ethics)

The remaining participants who clearly disagreed with the statement reported that it is not possible to draw recommendations from data gathered from a population. There is a danger of committing the is-ought fallacy: “Because I don't think the Is does imply the Ought. So, you may drive normative recommendations from what you learn, but I don't think they [researchers] draw them” (P3, empirical researcher in ethics). These participants talked about the difficulty of justifying how the empirical data allows one to come to a normative recommendation. Skeptical voices strongly emphasized the need to cultivate critical reflection on the normative significance of the empirical results:

“One of the things that empirical ethics doesn't do well is reflect critically on the results and the implications of the results. There is an assumption that if we are going to consult especially a population, that whatever they say has to be implemented, because that's the right thing to do. So, I'm not sure that we're at that stage in developing empirical bioethics.” (P15, empirical researcher in ethics)

### Developing and justifying moral principles

The use of empirical results for the development and justification of moral principles was highly contested. Participants considered this objective acceptable, but its acceptance was not always based on the practice and experience. For example, a participant noted, “I think you could… I have no objection to use empirical methods to do that either.” (P1, social scientist working on ethical topic) Others underlined that the objective of empirical research to justify moral principles depends on particular conceptions of justification, specific to moral pragmatics or experimental bioethics:

“If you are doing something like experimental bioethics, I think you might be interested. If a given moral principle is meant to be based on shared moral intuitions, and then you want to say that the moral intuitions are warranted or are responsive to the right kind of factors - or something like that - you might do that as a way of showing that the moral principles that are built on intuitions are justified”. (P9, empirical ethicist)

Several participants who disagreed with the statement, took issue with the term “justify”. They felt that empirical work can help refine, adapt, inform or find evidence to support moral principles nonetheless not to justify them because of the is-ought problem. One succinctly put it, “You can't justify moral principles by numbers or by practices.” (P20, empirical researcher in ethics).

Others who took issue with the term “justification”, accepted a negative objective of using empirical research to criticize moral principles: “You can perhaps see, if some moral principles cause troubles in practice and that leads to question them.” (P4, empirical ethicist) One participant claimed that refining or criticizing moral principles by means of empirical research is limited to concrete normative standards:

“I think empirical research can contribute to a critical evaluation of moral principles, particularly of mid-level moral principles, not the highest principles of many theories for example in Kant’s theory … because it has another origin …”. (P11, empirical ethicist)

A few participants pointed out that empirical results change depending on the context, so it is hard to make the kind of generalizations that is needed to support moral principles:

“The drawing on normative recommendations I guess is more where I would go, rather than justifying moral principles. Ehm...I think that will be tricky from an empirical ethics study to be able to then to just use that to justify specific moral principles, because the context would be so specific. I don't know if it's generalizable from any empirical ethics study.” (P21, empirical researcher in ethics)

### Identifying theoretical ethical issues

A majority of the participants accepted that empirical research can identify theoretical ethical issues. According to several participants, empirical research could reveal conceptual issues and challenge the coherence of theoretical frameworks: “And during that study I found a really interesting theoretical ethical issue and I bring it up in the paper: how do we actually define aggressive intervention?”. (P10, empirical researcher in ethics) “Sure! I think so. Yeah, you might find out that there are some interesting theoretical puzzles that you haven't realized until you went looked at the situation in a systematic way.” (P9, empirical ethicist) Another participant, discussing the current pandemic and development of normative theory based on data collected from past epidemics and the current pandemic, stated:

“In public health, the scholarship around ethical theory or normative theory, it's really just been building over the last twenty years. And I am so interested to see how this last point [identify theoretical ethical issues] will be taken up in the context of Covid because so much of public health ethics theory kind of started after SARS, where they started quarantining people. They didn’t have, you know, sort of the right ethical frameworks to do it, and they collected data and people, you know.” (P8, social scientist working on ethical topic)

Several participants felt it could be an objective of ERiB despite their own lack of experience with it: “I'm not sure I've ever done that. But it might also be beyond kind of my expertise to really go in theoretical concepts. So, I don't see why it couldn't be. I don't see why empirical ethics couldn't be useful for something like that.” (P15, empirical researcher in ethics).

A few participants who thought theoretical ethical issues could not be identified using empirical research stated that those issues are situated at a level of generality which makes it difficult to bridge with empirical research. One participant rejected this objective claiming that bioethics should remain a practical endeavor.

“I guess the link between the empirical research and the action of the philosopher here gets further and further away. There is more distance between empirical research and theoretical ethical issues than there was in 'understanding the context'… Ehm...I doubt if the really theoretical ethicists do use empirical research methods.” (P19, empirical researcher in ethics)

“I want to give an account of bioethics, that is practical in nature … , And that draws an important difference between bioethics and applied ethics …. But the answer got, it’s got to be no it seems to me. We can’t use empirical research to identify theoretical ethical issues in bioethics.” (P6, empirical ethicist)

### Empirical research as a source of morality to build new normative principles, rules or regulations

A majority of the participants thought that empirical research in bioethics could be a source of morality. Several participants considered it a motivating objective: “That's the one that I'm most excited about to be honest. I think that the ability of empirical ethics to do that type of innovation is probably not given enough due respect” (P15, empirical researcher in ethics). Another invoked feminist ethics to illustrate how empirical research can stimulate ethical innovations:

“Yes. Absolutely. I'm just I think... I'm just wondering... You know, I don't really know my history of ethics here, but some of the feminist theories around ethics of care and that sort of thing might have emerged in that way. And creating a sort of normative principle and all those regulations around the ethics of care. I think that has sort of emerged largely form some empirical research”. (P1, social scientist working on ethical topic)

One participant claimed that empirical research can be a source of morality by giving a voice to those who are unheard and have a rich experience:

“The experiences of those living the medical situations, living the diseases and living the therapies … , they are part of this reflective process, if they are asked and qualitative empirical research gives them a voice. … So, it's a way of being heard for those, who have very important insights from the field to deliver into the reflective process, that leads to an improvement also of principles, rules and regulations.” (P4, empirical ethicist)

Participants who did not think that empirical research could be a source of morality, stated “that’s too strong a claim for me.” (P6, empirical ethicist) Morality was not something “out there” waiting to be found: “… that morality is sourced out there, somewhere in the world and we find it, we find it by going to ask some people, doing or survey or something. I don’t hold that view” (P6). Others noted that empirical research could not be a “source of morality” but thought of empirical research as a source of information that is relevant for developing new rules and regulations or that provides a critical check for how moral rules work. One participant highlighted that empirical research needs to be extensive and consolidated if it should have an impact on inspiring sources of morality.

“Policy makers might want to be informed of that kind of moral attitudes, how they spread across the globe. And I think that CAN be interesting, indeed. But...not so much theoretically, so that it doesn't really tell you what is right or wrong. But in terms of how to design policies and laws - you know - how to educate the public before you implement something”. (P23, theoretical ethicist)

“I don't think you can do one empirical study and have it be generalizable. I think it needs to be critiqued … analysed … tested … reapplied …. You know, you can't just do an empirical ethics study and then say: "Okay, this is now a new - you know - normative way of doing things ... across the board".” (P21, empirical researcher in bioethics)

## Discussion

To date, scholars in the field have deduced the objective of ERiB by analyzing empirical work that have been carried out and how they are used by scholars in the field [14, 15, 18, 42]. Our study is the first one to provide data on whether researchers in the field identify with delineated purposes of ERiB, and to examine where their acceptability of such purposes changes and why. Our results reveal novel areas of agreement and disagreement to the eight objectives of ERiB that we proposed to researchers working in bioethics. The variation we found reflected differences of emphasis on the usefulness of empirical research for bioethics, but not substantial disagreements. On the one hand, the lack of substantial disagreement encourages an optimistic picture about interdisciplinary collaboration. On the other hand, even small differences of emphasis can have significant effects on how well researchers with different backgrounds can understand each other. Uncovering and analyzing these differences can improve interdisciplinary dialogue.

Overall, the participants supported a wide range of objectives for ERiB, albeit with varying enthusiasm. Objectives that contained the lowest ambition relating to normative implications gathered unanimous agreement, while more ambitious objectives were less endorsed. This is not surprising as many studies done in the field are descriptive, gathering information about the context, identifying ethical issues, and capturing attitudes of study participants [2, 48]. However, many participants, particularly those who described themselves as social scientists and empirical researchers in bioethics, accepted in principle the more ambitious potential objectives of empirical research, though admitting that they never did such kind of work. In light of their lack of experience, it is possible that participants felt that others could or might be doing such work and hence, did not disagree with those statements. Although we expected more participants to bring forth the is-ought gap as reason for hindering the integration of normative and empirical [2], only a few participants, mainly those who were empirical bioethicists, brought up the is-ought gap to express their disagreement with objectives that were more normative in nature.

All participants accepted the lowest ambitious objective: identification of ethical issues in practice. It was not viewed as an important objective mainly because empirical research was assumed to be hypothesis driven, in line with experimental strands of empirical bioethics [16, 19, 21]. Therefore, this objective was not considered a driving force of empirical research. However, not being very open to identifying ethical issues in practice is in tension with the promise of empirical bioethics that empirical research discovers bioethical issues in their authentic form as experienced by stakeholders ([7], p. ix).

When slightly more ambitious objectives for empirical research were presented to the participants, there was more reluctance to accept them. This was the case for the objectives ‘to find moral attitudes and reasoning patterns’, and ‘to evaluate how an ethical standard has been implemented’. Participants raised a definitional debate of whether these potential purposes should be called empirical bioethics, an ethical inquiry that aims to integrate normative and empirical research in a symbiotic manner [7, 49]. These two objectives were considered merely descriptive and in no need of ethics expertise. Intriguingly, all participants accepted the objective ‘understanding of the context of the phenomenon under study’ and considered it very important, despite the fact that it also does not include a normative goal in and of itself. There seems to be a misplaced aversion against the objective of ‘finding moral attitudes and reasoning patterns’, most probably because it is associated with doing simple surveys. The questionable practice of doing ‘ethics by opinion polls’ should not distract us from the importance of exploring people’s moral attitudes, as it is a significant part of contextual understanding [17].

The most ambitious objectives for ERiB were the most contested, that is, ‘striving to draw normative recommendations’, ‘developing and justifying moral principles’, and ‘identify theoretical ethical issues’. These objectives closely connect empirical research and normative implications, bringing forth the is-ought gap, which was a concern for the researchers with a philosophical-normative background. They wanted to critically reflect on the evaluative implications of empirical results. Salloch et al. [50] point out that there is always a risk of making simplistic or ill-grounded recommendations based on the empirical output when empirical researchers are not familiar with the methodological intricacies of normative analysis. However, other scholars have argued previously that social sciences should have a more important role in bioethical inquiry which goes beyond the production of empirical results [39, 40, 42, 43, 51]. As bioethics becomes more interdisciplinary, many questions about how to carry out such integration as well as quality standards of such an integration remain disputed [21, 52–56].

Other participants were reluctant to acknowledge directly the more ambitious objectives, whilst accepting and even supporting an indirect normative use of empirical research data. This means that the participants considered it possible for empirical data to ‘inform’ processes that would result in the drawing normative conclusions or recommendations and/or the justification of normative claims. The purpose of ‘informing’ was considered less problematic. However, it is important to question the perceived difference between the objective of informing and the objective of drawing or even the objective of justifying. The strong wording of ‘drawing’ and ‘justifying’ may have functioned as a psychological trigger for accusations of is-ought fallacy. Even if researchers frame their task in terms of how empirical research informs normative recommendations, the methodological issues of bridging the is and the ought do not disappear. We should be aware that the language of ‘informing’ can insinuate a free pass from engaging with the methodological complications of empirical bioethics because of it neutral and modest aura.

A contrast emerged between how participants viewed the potential of empirical research to identify ethical issues versus theoretical ethical issues in a practice. While the former statement was supported by all participants, the latter was among the most contested objectives. This hints to the assumption that empirical research in bioethics is mostly practice oriented. At the same time, debates in bioethics often involve controversies about how to define concepts, what concepts are relevant or appropriate [57–59], and for that identifying theoretical issues is also needed. Empirical work on theoretical goals is increasingly being carried out in the field of experimental philosophy (x-phi) and experimental bioethics (bio-xphi) [19, 20, 60–64]. Drawing on x-phi and bio-xphi literature, there are pleas to use of empirical methods to inform our understanding of concepts like health and disease [65, 66].

The objective of using of empirical research as a source of morality is highly ambitious and has not been explicitly stated in the literature. Nevertheless, the majority of participants were willing to endorse it. Empirical research could innovate our ethical thinking by giving a voice to unheard stakeholders and to those who have a rich moral experience. To illustrate this purpose, our findings alluded to the groundbreaking work of Carol Gilligan [67, 68] on women's conceptions of morality. Gilligan’s empirical research inspired the development of care ethics, which applies especially in nursing contexts [69, 70]. Empirical research can further lead to innovation in bioethics, but this kind of work is fraught with conceptual challenges. For instance, in the case of care ethics, the notion of caring has not been cleared enough, leaving the approach too vague [71, 72]. So, we still need a sophisticated conceptual analysis to guide empirically inspired innovation. This especially is true when new and exciting empirical data is presented as having significant normative implications. After the initial hype tempers, philosophical analysis can show that the normative implications of empirical findings are limited and unclear [73–75]. Unlike philosophers who focus only on abstract issues, empirically oriented ethicists are in a better position to assess the value and limits of empirical data. Thus, empirical ethics can harness this potential to mature into a field that not only opens bioethical issues to empirical study, but also critically reflects on the significance of empirical data.

### Limitations

Our findings are not generalizable. Other scholars working in the field of bioethics could have different opinions about what counts as an objective of empirical research in bioethics. We may thus have missed important voices and opinions. However, our sample of scholars was carefully selected to ensure unbiased sampling as well as to capture voices of scholars from all range of experience levels using random sampling. During the interviews, participants’ views were questioned and elicited using a tailored method that allowed us to confront them with views present in the literature. Whilst such an approach could lead to confirmation bias in a lay sample, the participants in this study were experts, invited not only to give but also to explain and argue their views. Although asking open-ended questions could have led to some of the objectives noted in the literature, doing so may not have allowed us to find nuanced findings, such as, disagreement that were terminology based. Acquiescence and social desirability effects were further prevented by the interviewer’s Socratic questioning and probing. This approach enabled us to access a wide range of responses and reasoning behind participants’ responses.

## Conclusion: accumulating experience in ambitious ERiB

So far, how empirical research is used in bioethics and what their objectives are have been derived theoretically by scholars observing the works done in the field [14, 15, 18, 42, 76]. This is the first qualitative study to explore researchers’ views on what are acceptable objectives of ERiB, and the reasoning for their positions. What transpired from our exploration is an overall shared enthusiasm for empirical work in bioethics, underscoring its increasing relevance in the field [2, 48]. At the core of disagreements about ambitious objectives of ERiB was the problem of integration of empirical insights in normative argumentation described as the bridging of two products of a different nature. Even scholars who exposed these difficulties shared an enthusiasm about the more ambitious objectives: “source of morality to build new normative principles, rules or regulations”. The acceptability of more ambitious objectives for ERiB will boil down to finding firm ground for and agreed upon methodological steps for the integration of empirical facts with normative inquiry.

An enthusiasm about the potential impact of empirical research, rather than experience, facilitated the acceptability of the most ambitious objectives of using ERiB. Most of the empirical work done by the participants has focused on meeting empirical objectives and informing practical recommendations. At the same time, the beliefs that philosophical work about moral principles is too disconnected from empirical research, that empirical inquiry does not have much to say about theoretical ethical issues, and that empirical research should address directly practical issues, generated disagreement about the most ambitious objectives. We recommend researchers to form interdisciplinary teams that engage more with ambitious objectives like developing moral principles and finding new theoretical issues and sources of innovations in bioethical thinking. We cannot say in advance how disconnected from empirical knowledge is the philosophical work on moral principles and normative reasoning. Accumulating experience in this area is an important step forward to develop empirical bioethics. If we want to solve practical issues in bioethics, we must not lose sight that they depend also on ethical theorizing and that ethical theorizing is subject to empirical scrutiny.

## Supplementary Information

**Additional file 1.** Interview Guide

## Data Availability

The datasets generated and analysed during the current study are not publicly available due to confidentiality and privacy reasons: the transcripts of the interviews contain information from which the identity of the participant can be easily retraced, thus we could include in the manuscript only excerpts of the raw material to support the findings and conclusions, along with the outline of the interview questions. However, the datasets are available from the corresponding author on reasonable request.
