# Supplementary material for: Acceptable objectives of empirical research in bioethics: a qualitative exploration of researchers’ views
Source: BMC Med Ethics. 2022 Dec 28;23:140. doi: 10.1186/s12910-022-00845-1 (PMC9794471; doi:10.1186/s12910-022-00845-1)
Supplement: Supplementary file 1 — Additional file 1. Interview Guide [file 12910_2022_845_MOESM1_ESM.docx]

**Supplementary File 1 submitted for manuscript “Acceptable objectives of empirical research in bioethics: a qualitative exploration of researchers’ views”**

**Interview Guide**

1. **Participants’ experience**
   1. Could we start with your research work (or scholarship) in the field of bioethics?
   2. Based on your empirical project(s) in bioethics, I would like to ask a few specific questions:
      - How did you design your most recent research project in empirical research in bioethics?
      - Could you explain how that project qualifies as an “empirical research in bioethics”?
      - How did you distinguish the empirical and the normative part of the study when writing the outputs?
2. **General questions**
3. In empirical research in bioethics, the project may involve both normative and empirical work. When you carry out such projects,
   - How do you work with these two poles of enquiry?
   - Could you describe the point in the project where do you feel that you need to focus on empirical or when you need to focus on normative?

2. I am now going to present different statements that relays the purpose of empirical research for bioethics. Thinking about bioethics field IN GENERAL, could we discuss your opinion on each one of them?

Statements: The purpose of empirical research in bioethics is …

1. Understand the context of the phenomenon under study
2. Identity ethical issues in practice
3. Find actual moral attitudes and reasoning patterns relevant to a practice
4. Evaluate how an ethical recommendation has been implemented
5. Draw normative recommendations
6. Develop and justify moral principles
7. Identify theoretical ethical issues
8. Source of morality to build new normative principles, rules or regulations
9. **Opinion and Views: Integrating normative and empirical**
   1. How did you carry out the integration of normative and the empirical OR how do you imagine it could be done in various contexts?
   2. What is more important in bioethics: normative analysis or empirical inquiry or is it context dependent? Please explain.
   3. What are the benefits of integrating normative work with empirical data?
   4. What are the difficulties of doing such integration?
   5. Scholars in bioethics have suggested that the trend towards empirical research in bioethics is leading bioethics away from normative work. What is your opinion about this?
10. **Closing**
    1. What would be your recommendation to this developing sub-field of bioethics?
    2. Would you like to add anything on the topic that you think we forgot to ask?

THANK YOU!
